# Remarks on the Poisonous Properties of the Laburnum

**Published:** 1829-08

**Authors:** A. T. Thomson

**Affiliations:** Professor of Materia Medica at the London University.


THE LONDON
Medical and Physical Journal.

Nº 366, VOL. LXII.] AUGUST, 1829. [Nº 38, *New Series.*

For many fortunate discoveries in medicine, and for the detection of numerous errors, the world is indebted to the rapid circulation of Monthly Journals; and there never existed any work, to which the Faculty, in Europe and America, were under deeper obligations than to the *Medical and Physical Journal of London*, now forming a long, but an invaluable series.—RUSH.

### ORIGINAL PAPERS, AND CASES,
### OBTAINED FROM PUBLIC INSTITUTIONS AND OTHER AUTHENTIC SOURCES.

### THE LABURNUM.

*Remarks on the Poisonous Properties of the Laburnum.* By A. T. THOMSON, M.D. Professor of Materia Medica at the London University. (In a Letter to Mr. NORTH.)

MY DEAR SIR,

IN the last Number of the London Medical and Physical Journal, a case of poisoning by the flowers of the Laburnum is detailed. This may appear strange to many who believe that the natural order Leguminosæ, to which the laburnum belongs, contains no poisonous plants, as it supplies a large proportion of the vegetable nourishment of man and other animals. The Laburnum is, nevertheless, a poisonous plant; and the active principle on which this quality depends resides both in the flowers and the seeds.

The Laburnum is a species of the genus CYTISUS, a family of plants belonging to the class *Diadelphia,* and the order *Decandria* of that class, in the Linnean system; and, as I have already mentioned, to the natural order *Leguminosæ* of Jussieu. According to Pliny,* the term *Cytisus* is derived from the name of an island of the Archipelago, whence the first species of the family described was procured. The Cytisus *laburnum* is a native of Helvetia; but it has long been cultivated in England as an ornamental plant: its long, pendulous racemes of bright-yellow papilionaceous flowers forming a beautiful appendage to the

* Liv. 13, c. 14.

delicate green of its ternate leaves, which are scarcely fully expanded when the blossoms are in their highest perfection, early in summer. The flowers are succeeded by long, narrow, many-seeded pods.

The vulgar have always regarded the seeds of the Laburnum as poisonous; and I recollect, as a boy, that I was warned against eating them. It is to MM. CHEVALLIER and LASSAIGNE that we are indebted for the knowledge of the poisonous principle of the Laburnum. These chemists, having analysed the seeds, procured an extract, which they named *Cytisine*, and which, on being taken into the stomach, operates as a violent emetic and purgative. A dose of five grains acts as severely as a dose of three grains of tartar emetic. M. Chevallier took eight grains, which operated in the most alarming manner; but the effects were combated successfully by means of acidulated drinks, freely administered: to use M. Chevallier's words, "par l'emploi de la limonade tartrique prise en grande quantité."

Cytisine has the appearance of an extract of a yellow colour; its taste is bitter and nauseous; it attracts moisture from the atmosphere; dissolves with difficulty in strong alcohol, very readily in weak alcohol; it is insoluble in ether, soluble in water. Its aqueous solution produces no effect on the colour of either litmus or turmeric; it is not precipitated by acetate of lead, the nitrates of silver and of mercury, the sulphates of copper and of iron, or by the hydrochlorates of barytes, lime, strontian, and tin.

These negative qualities, if they do not throw much light on the chemical nature of cytisine, show that it is a distinct principle; and the experience of its effects on the animal economy lead to the supposition that, like many other poisons, it may be made available as a remedial agent. As soon as the seeds are ripe, I shall order some cytisine to be prepared in the laboratory of materia medica of the university; and I will be most happy to enable you and my other professional friends to verify the results respecting its properties that have been obtained by the continental physicians.

As some of your readers may wish to prepare it for themselves, I subjoin the method of making it employed by MM. Chevallier and Lassaigne:

"Bruise the seeds of laburnum, digest them repeatedly in alcohol; and, having evaporated the tincture to the consistence of an extract, dissolve in water, filter the solution, and treat this with acetate of lead, to rid it of the acids and colouring matter. Filter the solution. The cytisine, mixed with acetate of lead in excess, passes through the filter; separate the salt of lead by means of sulphuretted hydrogen gas, and filter. The cytisine is procured in the form of an extract by evaporating the filtered solution."

Believe me, my dear sir, yours faithfully,

ANTHONY TODD THOMSON.

*3, Hinde street; 18th July,* 1829.

The case to which Dr. THOMSON refers in his letter occurred in our own practice, and we confess we were not aware at the time that the *flowers* of the Laburnum possessed any injurious properties. We mentioned the fact to two eminent botanists, both of whom stated that *none* of the leguminous tribe of plants were poisonous. The symptoms of depression produced in the child whose case we briefly related in our last Number, page 86, they were inclined to attribute to excessive nausea from an overloaded stomach. From Dr. Thomson alone could we obtain any information upon the subject. The following extract, which we take from the *Dict. des Sciences Med.* t. xlv. p. 187, confirms the statement with which he has favored us:

" *Cytisine* exists not only in the seeds of the *cytisus laburnum*, but, as it appears, in many other leguminous plants. It has also been recently detected in the flowers of the *arnica montana*; the emetic powers of which seem to depend more upon this poisonous principle than upon the larves of insects which have been mentioned by M. LEMERCIER. *Cytisine*, given in very small doses to different animals, produces vomiting, convulsions, and death. In man, in a dose of eight grains, besides very obstinate vomiting, it has caused vertigo, powerful spasmodic contractions, elevation of the pulse, and discoloration of the face. These symptoms lasted two days, and were followed by great depression, which continued, although in a diminished degree, for upwards of a fortnight."—EDITORS.

